# Patients’ confidence in coping with arthritis after nurse-led education; a qualitative study

**DOI:** 10.1186/s12912-016-0150-x

**Published:** 2016-05-04

**Authors:** Kjersti Grønning, Live Midttun, Aslak Steinsbekk

**Affiliations:** Department of Nursing Science and Center for Health Promotion Research, Norwegian University of Science and Technology (NTNU), Trondheim, 7491 Norway; Rauma Medical Center, Åndalsnes, Norway; Department of Public Health and General Practice, Norwegian University of Science and Technology (NTNU), Trondheim, 7491 Norway

## Abstract

**Background:**

The aim of this study was to explore *how* patients with chronic inflammatory polyarthritis described coping with their disease after a nurse-led patient education program and compare these experiences to patients in a control group who did not receive any education.

**Methods:**

This was a qualitative study nested within a randomized controlled trial (RCT) investigating the effect of nurse-led patient education for patients with chronic inflammatory polyarthritis. Twenty-six individual face-to-face interviews, 15 in the intervention group and 11 in the control group were conducted approximately two months after the educational program. The same opening question; *«Can you please tell me how you have been these last four months, since last time we spoke»,* followed by questions about the informants’ experiences of coping with disease-related challenges, disease activity changes, coping with disease activity changes, the informants’ perceptions of good and challenging situations to be in were asked to all informants.

**Results:**

Informants who attended the educational program expressed a strengthened confidence in coping with the consequences of having arthritis, which made them feel good. The strengthened confidence was attributed to sharing experiences with other participants in the group and learning something new. Informants in the intervention group further linked their confidence to 1) coping with disease fluctuations, 2) changed health behaviours and 3) knowledge about medications.

**Conclusions:**

Patients taking part in nurse-led patient education described a strengthened confidence in coping with their arthritis stemming from sharing experiences with other patients and learning something new.

**Trial registration:**

The RCT was registered in ClinicalTrials.gov (NCT00623922) in February 2008.

## Background

Rheumatoid arthritis (RA) and Psoriatic arthritis (PsA) are chronic inflammatory joint diseases characterized with various symptoms such as joint stiffness, pain, physical limitations and fatigue [1–3]. Coping with arthritis may be challenging, especially when new flares arise [4]. One approach to ensure that patients have sufficient skills and knowledge to cope with their diseases is to provide patient education [5], often built on theories or theoretical models that focus on patients’ resources, possibilities, coping and health behaviors [6–9]. This study chose Lazarus and Folkman’s definition of coping as “*constantly changing cognitive and behavioral efforts to manage specific external and/or internal demands that are appraised as taxing or exceeding the resources of the person”* ( [10], page 141). The definition is broad and integrates various approaches that patients may use to deal with “stressful situations” as living with chronic inflammatory arthritis may be [4].

Several studies of arthritis patient education have demonstrated beneficial effects [7, 11–13]. However, a limitation of quantitative evaluations of complex interventions, such as patient education, is that the chosen outcomes might not sufficiently assess the true impact of the intervention [14, 15]. Adding qualitative studies to such trials may give a better and deeper understanding of the effects. Qualitative interviews have shown that patients find it easier to accept their limitations and problems after attendance in patient education [16, 17]. Studies have also found that patients participating in patient education have increased their knowledge about their conditions [17] and succeeded in changing health behaviors [17, 18].

One way of investigating how taking part in educational interventions may influence patients’ lives is to compare changes in two groups, preferably within a randomized controlled trial (RCT) [19]. To the best of our knowledge, there are no such studies in patients with arthritis. To gain more knowledge about the process that patients participating in patient education go through, a qualitative study was nested within an RCT studying the effect of patient education. The RCT found beneficial effects on patients’ self-efficacy and well-being [13].

Therefore, the aim of this study was to explore *how* patients with chronic inflammatory polyarthritis described coping with their disease shortly after they had completed a nurse-led patient education program and compare these descriptions to patients in a control group who did not receive any education.

## Methods

### Design and data collection

This qualitative study was nested within a RCT [13] investigating the effect of nurse-led patient education. The RCT consisted of 141 patients with chronic inflammatory joint diseases (RA, PsA or unspecified polyarthritis). To get a qualitative sample of patients with different coping experiences, the last 26 patients included in the RCT representing patients of both genders, at different ages, different diagnoses and disease duration were interviewed. The informants had been interviewed once before, when they were randomized to the intervention (IG) or control group (CG). The results from these interviews are published [4]. The interviews lasted from 20 to 60 minutes and were conducted from September to October 2009. In order to make the interviews as similar as possible in both groups, no specific questions regarding the educational program were asked if not the informants brought this up themselves. The same opening question was asked to all informants followed by questions regarding the informants’ experiences of coping with disease-related challenges, if they had experienced any changes in their arthritis and how they coped with these changes, how the arthritis influenced their lives and if they could elaborate good and challenging situations to be in. The interview guide is shown below.

Interview-guide

Opening question:

«Can you please tell me how you have been these last four months, since last time we spoke? »

Follow-up questions:

- How are you coping with disease-related challenges now?
  - In your everyday life at home (with family and children)
  - At work (if the person was employed)
  - In your social life /leisure time
- Have you perceived any changes since last interview?
  - Disease-related changes
  - Coping (practical solutions /emotional reactions or thoughts)
  - Life quality in general (mentally, socially, physically), diet, habits
- Could you elaborate good situations to be in, why were these situations good?
- Have you experienced challenging situations, why were these situations challenging?

A detailed description of the nurse-led patient education intervention, which 15 of the informants participated in, is published [13]. However, a brief description of the intervention is given here. The intervention consisted of a combination of group and individual education. The individual session took place one or two weeks after the group sessions. The group sessions lasted three hours every other week over a period for six weeks. The intervention covered themes as arthritis symptoms, symptom circle, prognosis, problem solving, self-management, living with arthritis, motivation, goal setting, medical treatment options (effects, side effects), healthy life styles and community resources.

#### Analysis

The interviews were tape-recorded, transcribed verbatim and analyzed using the method of systematic text condensation (STC) [20]. STC is a modification of Giorgi’s phenomenological method where the essential steps are to get a sense of the whole material, to discriminate meaning units, to transform and abstract meaning units, and to synthesize the meaning units into consistent statements [20]. This strategy involves a process of de-contextualization (coding of meaning units and investigating the units more closely along with other meaning units that enlighten similar issues) followed by re-contextualization. In the process of re-contextualization, it is essential to make sure that the patterns still agree with the context from which they were collected [20]. The interviews from the control group were analyzed first. All transcripts were read to get a total impression and to identify the preliminary themes. The preliminary themes were refined after re-reading the interviews more thoroughly. Then, meaningful text units were coded and gathered into categories. The interviews from the intervention group were then analyzed. The analysis focused on deviations and comparisons on how the informants described to cope with the consequences of living with arthritis. The categories were refined and the analysis validated through detailed reading of the interviews and discussions between the authors.

## Results

Twenty-eight persons were invited and 26 agreed to participate. Table 1 presents the informants’ characteristics, showing a sample of 15 persons in the IG and 11 in the CG.

**Table 1:** Informant characteristics at baseline

Characteristics	IG (*N* = 15)	CG (*N* = 11)
*Gender*		
Females	12	10
Males	3	1
*Age*		
Mean age in yrs. (range)	58 (38–77)	58 (36–77)
*Living status*		
Living alone	1	1
Living with a partner	14	10
Children living at home	3	0
*Employment status*		
Employed	1	4
On disability benefits	11	4
Old-age pensioner	3	3
*Diagnosis*		
RA	9	10
PsA	4	1
UA	2	0
*Disease characteristics*		
Mean DAS28-3	3.2	3.1
Mean disease duration yrs. (range)	9 (1–19)	10 (2–23)
Using DMARDs	11	8
Using NSAIDs	4	2
*Coping*		
SE-other symptoms	66	74
SE-pain	56	66
PAM	73	70

*RA* Rheumatoid arthritis, *PsA* Psoriatic arthritis, *UA* Unspecified polyarthritis, *DAS28-3* disease activity score, calculated by C-reactive protein (CRP) and a 28 joint count (number of swollen and tender joints) [32, 33]. *DMARD* disease modifying anti rheumatic drugs, *NSAID* non-steroid anti-inflammatory drugs, *SE-other symptoms* self-efficacy other symptoms [34, 35], *SE-pain* self-efficacy pain [34, 35] and *PAM* patient activation measure [36, 37]

The main finding was that the informants in the IG expressed a strengthened confidence in coping with different consequences of having arthritis. Their strengthened confidence was linked to having shared experiences with other patients in the group and because the nurses and other health professionals had learned them something new. The additional findings are presented using the final categories as headings; 1) coping with disease fluctuations, 2) changed health behaviors and 3) knowledge about medications. Table 2 presents an overview of the findings.

**Table 2:** Overview of the findings

Research question	How do patients with arthritis perceive to cope with their arthritis after participating in patient education?	
Theme	Strengthened confidence	*“It [the sessions] was filled with a sense of belonging or connectedness. You have to know everything, things that were normal. You did not feel that you were the only one who felt it like this. [The interviewer]: Did it make you feel safer or more relaxed?*
		*I: Yes, it really did.*
		(Female, UA for seven years)
Categories	Coping with disease fluctuations	*“It felt good. I got confirmation about what I had thought and what I have been feeling, especially with the fatigue….Now I knew that this lack of energy is kind of a “side effect” of having arthritis”.*
		(Male, PsA for 15 years)
	Changed health behaviors	*“Yes, I have made changes. I have changed my diet. It is better now because we eat more vegetables”.*
		(Female, RA for seven years)
	Knowledge about medications	“*I always heard that it [cortizone] was dangerous. Especially from our family doctor; he was totally against it. I heard many horror stories. It was very informative and instructive to learn that it was not always that dangerous”.*
		(Female, PsA for 12 years)

*UA* Unspecified arthritis, *PsA* Psoriatic arthritis, *RA* Rheumatoid arthritis

### Strengthened confidence by sharing experiences and learning something new

When the informants in the IG talked about changes they had made, they linked these changes to exchanging disease-related experiences with other participants from the patient education program. This learning context made the informants conscious about their abilities and inherent resources to cope with the consequences of having arthritis. Informants from the IG described several situations where stories from other patients in the patient education program were helpful to them.

«*I think it was very positive with the course because you met others in the same situation. Then you kind of handle the disease differently, to put it that way».*

(Female from the IG who had lived with PsA for three years)

However, informants in both groups talked about the importance of having someone to talk to about being chronically ill to ease the burden of having arthritis. They preferred to talk to other people with arthritis or health professionals because they did not want to bother family or friends. Informants in the IG also explained that sharing personal stories about having arthritis made them discover that it was beneficial to be more open than they were before. It felt good to let others know how a life with arthritis could be like, and they could be honest about their limitations. They acknowledged their individual capacity, and that certain things were difficult to do. One man in the IG said that it finally felt good to tell the surroundings that he needed assistance with physical tasks because of the arthritis.

«*If someone asks: Can you lend me a hand? I just say that I cannot because I do not have a body for it (…) It is quite simple!».*

(Male from the IG, lived with RA for 13 years)

Informants in the CG did not express this confidence. They were reluctant about talking about limitations with others. Instead, they tried to demonstrate, non-verbally, that they were in pain or exhausted.

«*I don’t like to talk about being ill. I have pain, and that is life. You don’t need to talk to anyone about it».*

(Female from the CG, lived with RA for two years)

If the surroundings did not respond to their signals, they became a bit sad. In addition, informants in the CG who knew other patients with arthritis favored to talk to them about challenges instead of people without arthritis. Those who did not have anyone to talk to about being chronically ill expressed a wish for it.

### Coping with disease fluctuations

Informants in both groups explained how their fluctuating arthritis had influenced their everyday lives. However, informants in the IG and CG talked differently about coping with the fluctuations. Informants in the IG explicitly expressed that participating in the nurse-led patient education program made them more conscious of how the symptoms influenced their lives. They talked about the symptoms as a normal part of having arthritis, they were confident in coping with the symptoms, and they accepted that the arthritis would be a part of their future lives. Now they realized what kind of issues they needed to consider and what limits to set for themselves. Having this confidence made them feel good. They had finally realized that they were the only persons who really knew how living with arthritis could be. The informants in the IG described several situations where they had learned to say «no» and rather explain why they had to postpone or withdraw from demanding situations or activities. Having this confidence made them feel empowered and strengthened towards devaluating thoughts and comments from people who questioned if they were sick.

«*It was like some missing pieces fell into place (…) I don’t care about what others say anymore. We agreed at the course that we, who are sick, are the only ones who really know how we feel».*

(Female from the IG, lived with PsA for three years)

Stories from the informants in the CG were different. When they had to decline activities, invitations or stop doing things due to the arthritis, they felt sad and a bit depressed. They also felt guilty if they had to turn others down and rather avoided such situations or pushed themselves harder.

«*It is a bit difficult to handle that you are not able to do the things you used to do».*

(Female from the CG, lived with RA for two years)

However, a female IG participant reported a negative experience after the first session. She felt sicker, worried, anxious and subsequently, decided to withdraw from the rest of the program.

*«It was interesting in itself, but it was painful too, because I got worried (….) I started to check all the places that hurt. (…) Some of the others told me that I had a serious illness, and I just had to accept my situation. I felt my personality reduced to a disease. This was problematic for me, because I consider myself as much more than a disease. You need to look for possibilities and not limitations. Cultivating your disease is not good».*

(Female from the IG, lived with RA for 15 years)

### Changed health behaviors

Informants in the IG explained that they were enlightened after the educational program. They had become more aware of their opportunities, responsibilities, how to take action and how to make changes. These informants felt more confident about what to do, how to cope with certain challenges and how to solve problems related to their arthritis. They gave several examples of situations where they had made concrete changes. For instance, adjusting their daily schedule and prioritize differently to live a more social life, making diet changes to feel better, using new techniques for practical tasks, or starting to take medications. The changes had happened due to discussions with other patients and nurses during the patient education program. The informants seemed determined to continue with these actions and behaviors to maintain, attain or regain a perception of being of good health and prevent the arthritis from blushing.

«*I have become more conscious about reducing stress. We talked about such things in the course, and I think I manage this well. I kind of woke up».*

(Male from the IG, lived with RA for 13 years)

Informants in the CG talked mostly about changes they wanted to make, but something they did not do because it was difficult. Increasing their physical activity was a goal that several talked about, but did not accomplish.

«*[Interviewer]: You said you used to walk in the woods?*

*[Informant]: Yes, but I should have done it more often. I should have been better (…). My feet hurts, but I should have been better at walking more».*

(Female from the CG, lived with RA for three years)

Moreover, informants in the CG were also uncertain whether things were good or bad because they had heard and read contradictory things about how to behave.

«*I don’t know if it is healthy, but you need to eat some good stuff. I probably drink too much wine, but I like wine so I drink it. I have read a Swedish study that showed that people who drink a lot of red wine…. more than me (laugh)… have less disease activity».*

(Female from the CG, lived with RA for 16 years)

On the other hand, informants in the IG talked about a wide repertoire of self-management techniques they used in different situations. For instance, one man had up-taken relaxing techniques he had learned many years ago to reduce stress and pain because self-management techniques were discussed in the intervention. Regular use of these techniques had resulted in less pain and headache for this man.

«*This [the technique] is something I have done more consciously lately. We discussed such techniques in the meetings and I got reminded about stuff I used to know, but somehow had forgotten».*

(Male from the IG, lived with RA for 13 years)

Informants in the CG did not mention any changes of health behaviors or new techniques they had learned and carried out since last interview.

### Knowledge about medications

Informants in the IG and CG talked differently about knowledge related to medications. Both groups expressed a great fear of side effects. One man in the CG had stopped taking his medicines a long time ago because he felt uncomfortable. He was afraid of side effects, and needed more information of how the medicines could affect him. The information he had read on the internet scared him. Another informant in the CG said that he never took his prescribed medications because he felt insecure.

«*I need more information about my medicines before I dare taking them. I need to know more about how they work, how they will affect my body and such like».*

(Male from the CG, lived with PsA for two years)

Informants in the IG said that their understandings of medicines had changed after participating in the patient education program. They had become more confident about taking their medications as prescribed because they had gotten a deeper understanding of beneficial effects and more knowledge about possible side effects.

«*Before, I just took pills (…) Now, I pay more attention to them (..). I‘m more conscious and watchful and I have more respect for them [the medicines]».*

(Male from the IG, lived with RA for seven years)

## Discussion

The overall finding was that informants taking part in the patient education program expressed a strengthened confidence in coping with the consequences of having arthritis while informants in the CG did not express this confidence. Having met others with the same diseases in the patient education program promoted their confidence. The sessions facilitated for sharing experiences, exchanging thoughts, as well as getting knew knowledge or refreshing forgotten knowledge. This setting enhanced the informants’ confidence in coping with disease fluctuations, making favorable health behavior changes and taking medications as prescribed. The RCT, which nested this qualitative study [13], found that patients in the IG reported better well-being, self-efficacy and patient activation compared to the controls after four months. The findings from this qualitative study add a deeper understanding of the results from the RCT by exploring the participants’ experiences of how better well-being, self-efficacy and patient activation may be expressed through the informants’ voices.

### Strengthened confidence by sharing experiences and learning something new

The participants in the IG described that one of the most important issues in the nurse-led patient education program was meeting others with the same disease and sharing experiences with them. The exchange of experiences and knowledge from nurses, fellow patients and other healthcare professionals made them less worried when they experienced new disease flares. They knew that periods with increasing and fluctuating symptoms were normal when having arthritis. This finding is consistent with other qualitative studies suggesting that symptom validation in group education gives a legitimacy of symptoms and fears as normal [21]. Patient education may endorse participants with increased empowerment and control of their situation [18] through separating the disease from the person and consider the arthritis symptoms as challenges they can work on. However, one woman in the IG felt sicker by attending the patient education program and dropped out after the first session. This informant experienced the discussions on disease symptoms as targeting her weaknesses instead of aiming for her resources. For this informant, the group session was negative instead of empowering. Whether this person would have experienced the program differently if she had participated in the remaining sessions is unknown.

Nevertheless, other informants in the IG gave detailed descriptions about beneficial information and support from other patients and nurses during the sessions. Having the opportunities to express fears and thoughts with nurses and fellow patients were highlighted as important. Makelainen and colleagues [22] found that emotional support provided by nurses in patient education is important for patients’ overall well-being. However, nurses must keep in mind that negative patient experiences may occur when working with patients in groups. The experience from the woman dropping out of the program shows the importance of balancing the content and group-discussions between disease-related challenges, problems, and patients’ health and coping resources.

Furthermore, informants in the IG expressed increased personal integrity through setting limits for themselves, and caring less about healthy people’s opinions on whether they were sick or not. Barlow and colleagues [16] found that validation of symptoms through patient education contributed to patients’ acceptance of having a chronic disease. Patients find inspirational modeling, social validation, connectedness and comparison in patient education, as beneficial. Participants in the IG highlighted that participating in the patient education program improved their communication about having arthritis with others, which is in line with another study [18] showing increased communication skills after participating in patient education. That study found that patients being able to communicate and assert their needs to other people became understood and achieved support [18]. Social support is essential for patients with chronic diseases and a buffer against negative life quality [23].

### Changed health behaviors

Participants in the IG described several situations where they had taken a greater responsibility of their health. These findings are consistent with another study [18] showing that patients accept their disease as not curable but something they can actively work on to prevent negative impacts. Such changes in patients’ disease responses may be an expression of increased patient activation as shown in the RCT [13]. Patient activation is about patients’ abilities to undertake active roles as administrators of their health and health care. It further refers to patients’ understanding of their roles, knowledge, skills and confidence to manage their health [24]. Group settings may feel like a safe place to pursue changes with fellow members providing a credible source of persuasion, validation and legitimization of each other’s experiences [25]. The nurse-led patient education program intended to act as a safe arena for learning new strategies, trying new behaviors and making patients believing that they could obtain their goals. This study shows that the patient education program created a setting where the informants learned new things and increased their self-confidence, which do fit the pathway of self-efficacy [26] and patient activation [27].

### Knowledge about medications

Informants in the IG explained that they had become more conscious about taking their medication as prescribed and were less worried about side effects. Studies show that patients seem to calculate perceived risk of treatments against perceived benefits [28]. Adherent patients have stronger beliefs about the necessity of their medication than non-adherent patients do [29]. Our findings illustrate that patients’ knowledge influence patients’ beliefs and consciousness related to their medical treatment regimes, which underpins the importance that nurses discuss patients’ perceptions of medications in patient education programs. However, the results also showed that patient education might have side effects in terms of negative patient experiences. Knowledge about possible side effects are especially important for nurses in charge of patient education. Nurses need to be aware of how patients in groups respond to the discussions. It is essential to balance the content between patients’ challenges and symptoms with opportunities and coping resources.

### Strengths and limitations

Qualitative research is a systematic and reflective process where the transferability [30] and trustworthiness (credibility, dependability and transferability) of the findings ought to be discussed [31]. The credibility and dependability of the findings is good, as we explored experiences from patients taking part in nurse-led patient education in addition to patients in a control group. The face-to-face interviews gave detailed information about potential changes that may occur after attendance in patient education. The participants described various coping experiences and a range of perspectives that were highlighted with quotations from the informants. Another strength is the research group, consisting of persons with different academic perspectives, a senior year medical student, a nurse and a sociologist, the latter two with PhD. The findings are also transferable to other settings and patient populations as the characteristics of the informants are similar to other populations of patients with arthritis [2].

However, there are some noteworthy limitations. Selection bias might have influenced the findings due to the enrollment in the RCT. Patients not wanting to participate in an RCT may be different from those enrolled. It is possible that patients with good coping abilities declined participation because they did not perceive any needs for patient education. Patients with low coping abilities may also have declined participation because they could be embarrassed due to lack of skills. Nevertheless, the data indicate that the sample were heterogeneous comprising patients with different coping resources, men and women at different ages, and variations in disease duration and disease severity. We decided to include the experiences from one informant who dropped out of the intervention as these experiences illuminated an important perspective related to “possible side-effects” of the intervention.

It is possible that the interviewer could have asked some leading questions. However, we critically appraised the transcripts and only noticed a few deviations where the interviewer mentioned the patient education program before the informants started to talk about it themselves. Our preconceptions were that the informants would have different experiences, as we knew the results from the RCT. Nevertheless, we were extra vigilant throughout the analyses process by rechecking the transcript to verify that the differences were rooted in the data.

## Conclusion

This qualitative study found that patients benefit from participating in nurse-led patient education. The interaction with other patients drives the process of coping along with obtaining new knowledge to better deal with the consequences of having chronic inflammatory arthritis. The informants participating in the educational program described a newfound and strengthened confidence on how to cope with their arthritis. The experiences from the participants in the nurse-led patient education program were in concordance with the pathway of self-efficacy and patient activation, which many patient education programs are based on.

## Ethics and consent to participate

We conducted the study in accordance with the Declaration of Helsinki. The Regional Ethics Committee in Medicine, Central Norway approved the study (4.2007.2472). All participants invited to the study received oral and written information about the purpose of the study along with information about their right to withdraw without having to give any reason for their withdrawal. We obtained a written consent from all participants.

## Consent to publish

Not applicable.

## Availability of data and materials

All data supporting the findings in this article is contained within the manuscript.

## Abbreviations

CG: Control group
CRP: C-reactive protein
DAS28-3: Disease activity score
DMARD: Disease modifying anti rheumatic drugs
IG: Intervention group
NSAID: Non-steroid anti-inflammatory drugs
PAM: Patient activation measure
PsA: Psoriatic arthritis
RA: Rheumatoid arthritis
RCT: Randomized controlled trial
SE: Self-efficacy
STC: Systematic text condensation
UA: Unspecified arthritis
